# 

**Published:** 1861-09

**Authors:** 


### BOOKS RECEIVED FOR REVIEW.

*Burnstead on Venereal.*—The pathology and treatment of venereal diseases, including the results of recent investigations upon the subject, by Freeman J. Burnstead, M. D., lecturer upon venereal diseases at the College of Physicians and Surgeons, New York ; Surgeon to St. Luke's Hospital ; Assistant Surgeon to the New York Eye Infirmary, *with illustrations.*—Philadelphia : Blanchard & Lea.

*Barwell on Diseases of the Joints—Illustrated with Engravings.*—In one very handsome octavo volume of about 500 pages. Philadelphia: Blanchard & Lea.

*Fiske Fund Prize Essay.*—The morbid effects of the retention in the blood of the elements of the urinary secretions, by William Wallace Morland, M. D. Being the dissertation to which the Fiske Fund Prize was awarded, July 11, 1860. Philadelphia : Blanchard & Lea.

We shall take great pleasure in reviewing and giving notice of the above valuable works, as soon as time and space will permit.

*Transactions of the Illinois State Medical Society.*—Containing Medical and Surgical Reports of value ; also, the retiring President's Valedictory Address, by David Prince, M. D., of Jacksonville, Ill., and a popular lecture before the Illinois State Medical Society and the citizens of Paris, Edgar County, Illinois, upon the mutual relations and consequent mutual duties of the medical profession and the community.

*Transactions of State Medical Society of Indiana,* with valuable reports upon Medical Teaching, Medical Practice, Surgery, &c., &c., It has been received too late for further notice.

